# Perceived Nurse—Physician Communication in Patient Care and Associated Factors in Public Hospitals of Jimma Zone, South West Ethiopia: Cross Sectional Study

**DOI:** 10.1371/journal.pone.0162264

**Published:** 2016-09-15

**Authors:** Fikadu Balcha Hailu, Chanyalew Worku Kassahun, Mirkuzie Woldie Kerie

**Affiliations:** 1 Department of Nursing, Jimma University, Jimma, Ethiopia; 2 Department of Nursing, Mada Walabu University, Bale, Ethiopia; 3 Department of Health Economics, Management & Policy, Jimma University, Jimma, Ethiopia; Cardiff University, UNITED KINGDOM

## Abstract

**Background:**

Nurse–physician communication has been shown to have a significant impact on the job satisfaction and retention of staff. In areas where it has been studied, communication failure between nurses and physicians was found to be one of the leading causes of preventable patient injuries, complications, death and medical malpractice claims.

**Objective:**

The objective of this study is to determine perception of nurses and physicians towards nurse-physician communication in patient care and associated factors in public hospitals of Jimma zone, southwest Ethiopia.

**Methods:**

Institution based cross-sectional survey was conducted from March 10 to April 16, 2014 among 341 nurses and 168 physicians working in public hospitals in Jimma zone. Data was collected using a pre-tested self-administered questionnaire; entered into EpiData version 3.1 and exported to Statistical Package for Social Sciences (SPSS) version 16.0 for analysis. Factor analysis was carried out. Descriptive statistics, independent sample t-test, linear regression and one way analysis of variance were used. Variables with P-value < 0.05 were considered as statistically significant.

**Results:**

The response rate of the study was 91.55%. The mean perceived nurse-physician communication scores were 50.88±19.7% for perceived professional respect and satisfaction, and 48.52±19.7% for perceived openness and sharing of patient information on nurse-physician communication. Age, salary and organizational factors were statistically significant predictors for perceived respect and satisfaction. Whereas sex, working hospital, work attitude individual factors and organizational factors were significant predictors of perceived openness and sharing of patient information in nurse-physician communication during patient care.

**Conclusion:**

Perceived level of nurse-physician communication mean score was low among nurses than physicians and it is attention seeking gap. Hence, the finding of our study suggests the need for developing and implementing nurse-physician communication improvement strategies to solve communication mishaps in patient care.

## Introduction

Nurse-physician communication is more than just exchanging of information in which common understanding across health care team is established [1, 2]. It is described as a professional interaction, working together, shared decision making around health issues, formulating collaborative patient care plan in which the actual team’s performance is measured [3, 4]. To get the job done right, information need to be transferred in a clear and reliable way with respect and satisfaction. It is not only what is said that matters, but also the way it is communicated between nurse and physician [5].

Reasons for communication mishaps in patient care are of multifaceted. These include organizational factors, work attitude related individual factors and personal behavior related individual factors [6–9]. Workplace related reasons of communication mishaps include: organization’s culture, stressful environment, a culture of autonomy and hierarchy, a lack of team training and treatment plans [10], lack of inter-professional meetings [7], lack of accountability, lack of defined roles and responsibilities, mechanism of payment and rewards regarding clinical responsibility and differences in schedules [6, 8]. With regard to work schedule, working during the evening shift was found to lower openness of communication compared to day shift [9].

Among individual related factors age [7, 10], personal values and expectations, levels of educational preparation and qualifications were associated with ineffective communication [6, 8]. Similarly a qualitative study in USA and Belgium showed lack of nurses’ skill in assessment, time constraints, physician attitude towards the nurses, nurses’ attitude towards the physicians, way of communication, poor communication skills of nurses and physicians were individual related factors associated with nurse physician communication [11, 12].

Physicians’ behavior was found to be one of the major factors negatively affecting nurse-physician communication. Difficulty of nurses to talk with physicians when the physicians are hurried, unwillingness of physicians to deal with problems and consider nurses’ views, rudeness and disrespect behavior of physicians, interrupting nurses before finishing report and feeling of frustration after interaction with physicians were identified as factors affecting nurse-physician communication [13, 14]. Whereas, strong professional communication and respect were found to be a key to successful communication [14].

Inaccurately communicated and misunderstood information increases a tension among health care team members [15] and have been the focus of ongoing argument [5]. Rosenstein A, et al revealed that 30% of respondents knew at least one nurse who left the hospital as a result of disruptive physician behavior like raising the voice, disrespect, condescension, berating colleagues and patients, and use of abusive language that can be contributing factor to nurse satisfaction and morale [16].

Although nurses and physicians share a common agenda of caring for the sick the two professions fail to understand their complementary roles [3]. Dysfunctional nurse-physician communication is linked to medication error, medical mistakes, a major risk factor for unwanted preventable patient injury [9, 17–19], death, medical malpractice claims [6, 9, 19–22], delaying care and extending the length of a patient's stay[19].

Nurse-physician ineffective communication has an impact on nurses' and physicians’ job satisfaction, turnover, and above all the quality of care [9, 21, 23, 24]. When nurses and physicians are not communicating effectively, patient safety is at risk due to lack of critical information, misinterpretation of information, unclear orders and overlooked changes in status [8].

Nurse-physician communication can be conflictive that arise from competition for status and power, values and believes [5]. On the surface, there are important benefits from nurse- physician collaborative work, and yet this collaborative emphasis is not sufficiently stressed in medical education nor seen in actual practice [14].

Studies showed that physicians had better overall communication openness than nurses [9, 25, 26]. A study conducted in intensive care unit of West Indies University Hospital showed that overall communication openness was thought to be better among physicians (73%) than the nurses (32%). Most physicians (70–73%) thought doctor-to-nurse communication was good and less proportion of nurses (35–67%) felt less communication with physicians.

However there are also studies that revealed nurses have greater perception of communication than physicians. D. Tschannen and E. Lee revealed that nurses working in medical and surgical units with higher education levels, more years of experience and more positive environment had greater perceptions of communication openness with physician [9]. In line with this most of nurses (85%) found that taking advice from senior residents and higher percentage of physicians found that taking advice from senior nurses is easy [25]. It was revealed that mean score for sharing of patient information was higher for nurses than physicians and perceived communication was more open among nurses than physicians [9, 26]. Similarly, a study conducted in Egypt Alexandria Main University Hospital, showed that among dimensions of nurse-physician interprofessional relationships mean score of communica12tion among nurses was slightly higher (34.90 ±4.47) than among physicians (33.67±2.13) [27].

A study conducted in three teaching hospitals of Iran showed that female nurses and nurses with more than 20 years of experience had better perceived positive communication with physicians [28].

Overall more studies related to nurse-physician communication have been conducted in developed countries and in few of hospital units. Such studies are generally lacking in Africa and to our knowledge none in Ethiopia. In addition, different studies found that nurse-physician communication is a key in health care practice for improving health care delivery and outcomes of patients. In developing countries, including Ethiopia, health care delivery is not computerized and also not supported by different protocols guiding nurse-physician communication. Therefore, this study assessed the perception of nurses and physicians towards nurse-physician communication and associated factors. The output of this study may inform the Ethiopian Federal Ministry of Health and other relevant bodies to set specific strategies used to improve interprofessional communication.

## Conceptual Framework

**Fig 1:**
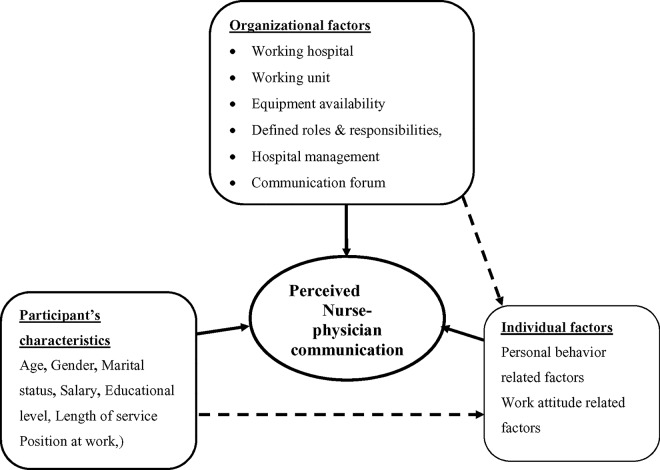
Conceptual framework: nurse-physician communication in patient care and associated factors in public hospitals of Jimma zone southwest, Ethiopia, 2014.

The conceptual framework was developed after reviewing relevant literatures (Fig 1).

## Methods and Materials

### Study area and period

A cross-sectional institution based study was conducted in three public hospitals of Jimma zone namely: Jimma University Teaching Hospital (JUTH), Shenen Gibe hospital and Limmu Genet hospital, which are situated at south west of Addis Ababa, the capital of Ethiopia at around 357–450 km. JUTH is the only teaching and referral hospital in the southwestern part of the country providing specialized clinical services to about 15 million people in the catchment area [29]. The study was conducted from March 10, 2014 –April 16, 2014.

All 433 nurses and 185 physicians (including residents) working in these three public hospitals were included in the study. To be included in the study nurses and physicians need to finish their probation period (who worked for at least six months) and available during data collection period in their respective hospitals.

### Instrument and measurement

Data was collected using pre-tested Likert scale type self-administered English version questionnaires which have 3 parts:

Part–I: Participants’ characteristics (age, gender, marital status, educational level, salary, position at work, length of service).

Part–II: Perception of nurses and physicians towards communication in patient care with 19 items and participants were asked to rate each item on a 5-point scale which ranges from never (1) to always (5).

Part–III: Perception of nurses and physicians towards factors associated with nurse-physician communication which has 16 items and participants were asked to rate each factor on a 5-point agreement scale which ranges from strongly disagree (1) to strongly agree (5).

Communication scales questionnaire was adapted and modified from a study conducted in Iran, psycho-metric properties of the nurse–physician collaboration scale used in Japan and nurse-physician communication scale used in long-term care setting used in Connecticut [13, 28, 30]. In this study two communication subscales were emerged following principal component analysis (PCA), named as professional respect and satisfaction with inter-item reliability of α = 0.901, and openness and sharing of patient information inter-item reliability of α = 0.91.

Moreover, we included questions on participants characteristics and nurse-physician communication factors after reviewing relevant literatures [27]. After we examined the 16 item scale factors associated with nurse-physician communication using exploratory factor analysis, three latent factors were emerged, named as 1) organizational factors (6 items) with reliability of α = 0.85, 2) work attitude related individual factors (6 items) with α = 0.83 and 3) personal behavior related individual factors (4 items) with item reliability of α = 0.75.

Questions were combined after testing for inter-item reliability using the Cronbach's alpha (which was α = 0.89 for communication scale items and α = 0.94 for communication factors items) score from the pretest data which was done in Woliso Saint Luke Hospital (around 150 Km away from Addis Ababa), making 5% of the study population, before the actual data collection. This was done in order to assess the contents, clarity, sequence and flow of the questionnaire.

The perceived communication scores were standardized as the percentage of the maximum scale (%SM) scores to facilitate comparison. This enables future researchers to easily compare their findings with those in this study even if they make use of different number of items and/or response categories. These scores lie between 0 and 100 [31, 32].

## Data collection procedures

The data collection was facilitated by five diploma level qualified nurses who were given a one day training to familiarize them on data collection procedure and instrument. Shift of the respondents was arranged in contact with shift leaders for nurses and department heads for physicians. The data collection facilitators distributed the self-administered questionnaires to the respondents to fill it out. In the absence of respondents repeated revisits were done.

### Operational definitions

- Work attitude related personal individual factors: some of these are noncompliance with advice, negligence of duty, abuse (verbal, physical and sexual), poor attitude to work, uncooperativeness at work, gender difference
- Personal behavior related individual factors: some of these are disruptive behaviors, unfavorable attitude toward other professionals (nurse or physician) and poor interpersonal communication skill
- Organizational factors: some of these are differential treatment of nurses or physicians in the hospital, absence of forum regarding nurse-physician communication, lack of shared vision between nurses and physicians in the hospital, malfunctioning of equipment in units, frequent supply shortage in units
- Perceived nurse-physician communication score: Measured by two subscales generated from an 18-item scale containing statements related to nurse-physician communication. The scales are named as perceived professional respect and satisfaction and perceived openness and sharing of patient information. The higher the score the higher perceived nurse-physician communication during patient care.
- Perceived professional respect and satisfaction score with nurse-physician communication: Nurse-physician communication subscale containing nine 5-point Likert scale items with minimum potential score of 9 and maximum potential score of 45. The mean percentage of the score was calculated using the following formula.

$$\%SM=\frac{Actual\;Score-Scale\;Minimum\;Score}{Scale\;Maximum\;Score-Scale\;Minimum\;Score}\times 100\%$$

The higher the score the better the professional respect and satisfaction with nurse-physician communication. These scores lie between 0 and 100 [31].

- Perceived openness and sharing of patient information score: Nurse-physician communication subscale containing nine 5-point Likert scale items with minimum potential score of 9 and maximum potential score of 45. Mean score was calculated in the same manner as above. And the higher the score indicates the better openness and sharing of patient information during nurse-physician communication in patient care. These scores lie between 0 and 100.
- Organizational factors score: perceived nurse-physician communication factor subscale generated through PCA and measured by six items with agreement Likert scale and has minimum potential score of 6 and maximum potential score of 30. The higher the score indicates the more to be nurse-physician communication factor.
- Personal behavior related individual factors score: perceived nurse-physician communication factor subscale generated PCA and measured by four items with agreement Likert scale and has minimum potential score of 4 and maximum potential score of 20. The higher the score indicates the more to be nurse-physician communication factor.
- Work attitude related individual factors score: perceived nurse-physician communication factor subscale generated through PCA and measured by six items with agreement Likert scale and has minimum potential score of 6 and maximum potential score of 30.The higher the score indicates the more to be nurse-physician communication factor.

### Data processing and analysis procedures

Data were checked for completeness, edited and entered into EpiData version 3.1 and exported to SPSS version 16.00 for analysis. The data were explored using descriptive statistics and frequencies for cleanliness. Scatter plots, skewness, and kurtosis were examined to determine the shape of the data distribution. On the basis of this information, data were determined to be fairly normally distributed, so no transformations were required.

To see factors that were considered and to generate common factors that reflect perceived nurse-physician communication score, PCA was implemented on the communication scale. Prior to performing PCA, the suitability of data for factor analysis was assessed. The results revealed the presence of many coefficients of 0.4 and above, Kaiser-Meyer-Olkin (KMO) measure of sampling adequacy was 0.94, and a Barlett's test of Sphericity (*P* <0.001). This indicates that sampling adequacy and the matrix were suitable to perform factor analysis. To assist in the decision concerning the number of factors to retain, the following criteria were used: 1) an Eigen values of one or more for each factor, 2) an item-to-factor loading of 0.4 or greater, 3) a minimum of three items loading on a factor; a factor with fewer than three items was considered weak and unstable, 4) Catell’s scree plot test which recommends retaining all factors above the elbow, or break in the plot, as these factors contribute the most to the explanation of the variance in the communication data set.

When the 19 communication scale items were entered into PCA three latent/proxy-variables were extracted, nine items have contained in each of the two components and only one item for component three. Because the third component has less than three items, it was discarded from the communication scale measurement items. Factor scores were created and used in the subsequent analysis. Following that, one-way analysis of variance (ANOVA) and independent sample t-tests were used for comparing perceived communication scores across the categories. Mean scores in the perceived nurse-physician communication were tested using a one-way between-groups analysis of variance (ANOVA) and post hoc comparisons using the Tukey honestly significant different (HSD) test to explore each group with regard to length of service category and educational qualification category to see in which category perceived nurse-physician communication difference was observed.

Bivariate analysis was done to see the independent effect of predictors and multiple linear regression analysis was conducted to identify final predictors of perceived communication after controlling for other independent variables. Variables with p ≤ 0.25 in bivariable analysis were entered in the final model. Participant’s characteristics, individual related factors and organizational factors were entered independently. For respect and satisfaction with communication, first participants’ characteristics were assessed while in the second organizational factors were included. For openness and sharing of patient information, first participant’s characteristics were entered, in the second and third model factors related to individual work attitude and organizational factor were included respectively.

Finally, variables with P ≤ 0.05 in the above models were entered to the final regression models. The assumptions of t-test, ANOVA and multiple linear regressions were checked. And finally, the result were summarized and presented in statements, tables and graphs.

### Data quality management

The quality of data was assured by pre-testing the questionnaire on 5% of the actual sample size in the aforementioned hospital one week before the actual data collection. Based on the pretest appropriate modifications of questionnaire were made. Moreover the data collectors were given one day training and data were checked for completeness every day. Those incomplete questionnaires were discarded during data entry. EPI DATA version 3.1 was used to minimize data entry errors. Proper categorization and coding were done during data cleaning.

### Ethical consideration

Before the actual work, ethical clearance and approval was obtained from the Institutional Review Board (IRB) of the College of Health Sciences, Jimma University. In addition permission was obtained from the respective hospitals. A letter of consent outlining the aim and giving further details about the study accompanied each questionnaire. To assure anonymity and confidentiality the names of the participants were replaced by codes. In addition, prior to administering the questionnaires, oral informed consent was obtained from the participants.

## Results

### Characteristics of the study participants

A total of 509 participants were involved in the study and of which 466 completely filled the questionnaires giving a response rate of 91.55%**. **Out of 466 study participants 67.2% (313) of them were nurses, 87.8% (409) of them were from referral hospital, 63.3% (295) were males and 55.4% (258) were singles. The participants’ age ranged from 21 to 58 years, with a mean age of 28.95 ± 6.82 years. The majority of the respondents were in the age group of 25 to 31 years.

Regarding educational qualification 42.1% (196) of nurses were diploma holders and 60.1% (92) physicians were residents at different level. The participants had work experience that ranges from half a year to 39 years with a mean of 5.57 ± 6.085 years. The participant’s monthly salary ranged from 1033 Ethiopian Birr (EBR) to 10200 EBR with a mean salary of 2824.72 ± 1738.55 EBR. Concerning positions currently held in the hospital, 60.1% (280) of nurses were staff while 17.6% (82) physicians were residents at different level and 24.9% (116) of the participants were working in OPD (Table 1).

**Table 1:** Characteristics of nurses and physicians working in public hospitals of Jimma Zone, Southwest Ethiopia, 2014 (n = 466).

Participant characteristics		No	%
**Professional category**	Nurse	313	67.2
	Physician	153	32.8
**Working hospital**	Teaching /referral hospital	409	87.8
	District /non-teaching Hospitals	57	12.2
**Sex**	Male	295	63.3
	Female	171	36.7
**Age category (years)**	18–24	107	23.0
	25–31	265	56.8
	32–38	49	10.5
	>38	45	9.7
**Marital status**	Single	258	55.4
	Ever married	208	44.6
**Educational Qualification**	Diploma nurse	196	42.1
	BSc nurse	117	25.1
	Resident physician	92	19.7
	Specialist and General practitioner (staff) physician	61	13.1
**Salary category (ETB)**	<1427	121	26.0
	1428–2250	171	36.7
	2251–3414.25	58	12.4
	>3414.26	116	24.9
**Position presently hold in the hospital**	Staff nurse	280	60.1
	Resident physician	82	17.6
	Clinical staff physician	52	11.1
	Head nurse	26	5.6
	Department head physician	13	2.8
	Supervisor nurse	4	0.9
	Lecturer physician	4	0.9
	Matron nurse	3	0.6
	Medical director physician	2	0.4
**Service year (in years)**	≤2	156	33.5
	3–5	184	39.5
	6–8	54	11.6
	9–11	20	4.3
	>11	52	11.2
**Working unit category**	Medical ward	67	14.4
	OPD	116	24.9
	Surgical ward	76	16.3
	Pediatrics ward	54	11.6
	Obstetrics and gynecology ward	52	11.2
	Chronic illness	25	5.4
	OR	25	5.4
	ICU	18	3.9
	Ophthalmology ward	16	3.4
	Psychiatry ward	9	1.9
	Others	8	1.7

### Perceived nurse-physician communication in patient care

#### Description of nurse-physician communication sub scale items

Regarding perceived professional respect and satisfaction communication subscale participants always or usually did not feel angry after nurse physician interaction only 33.5% of the time (7.5% + 26%); thus, the remaining 66.5% of the time felt angry at least sometime after such interaction. Similarly the participants reported that 67.6% (37.3% + 23.4% + 6.9%) of the time felt frustrated at least sometimes after nurse physician interaction. On the other hand, 36.5% (9.7% + 26.8%), 36.3% (8.4% + 27.9%), 31.7% (7.7% + 24%) and 28.1% (7.9% + 20.2%) of them always or usually felt understood, respected, pleased and satisfied respectively after nurse physician interaction. Only 18.9% (4.1% + 14.8%) and 26.8% (9.4% + 17.4%) of them always or usually felt understood equal and joyful after nurse physician interaction. Whereas, 52.1% (21.2% + 30.9%) of them always or usually felt they received correct information relevant to care given to patients during nurse-physician communication (Table 2).

**Table 2:** Frequency of perceived professional respect and satisfaction items during nurse-physician communication among nurses and physicians working in public Hospitals of Jimma Zone, Southwest Ethiopia, 2014 (n = 466).

Respect and satisfaction on communication subscale items (α = 0.90)	Always		Usually		Sometimes		Rarely		Never	
	No	%	No	%	No	%	No	%	No	%
Feeling not angry after nurse and physician interaction	35	7.5	121	26.0	180	38.6	111	23.8	19	4.1
Feeling not frustrated after nurse and physician interaction	37	7.9	114	24.5	174	37.3	109	23.4	32	6.9
Feeling understood after nurse and physician interaction	45	9.7	125	26.8	174	37.3	96	20.6	26	5.6
Feeling respected after nurse physician interaction	39	8.4	130	27.9	185	39.7	77	16.5	35	7.5
Feeling pleased after nurse physician interaction	36	7.7	112	24.0	177	38.0	106	22.7	35	7.5
Feeling satisfied after nurse physician interaction	37	7.9	94	20.2	167	35.8	119	25.5	49	10.5
Nurses and physicians have equal understanding during interaction	19	4.1	69	14.8	169	36.3	145	31.1	64	13.7
Talking between nurse and physician is joyful	44	9.4	81	17.4	172	36.9	103	22.1	66	14.2
Received correct information relevant to give care for the patient	99	21.2	144	30.9	143	30.7	53	11.4	27	5.8

Regarding perceived openness and sharing of information the participants reported that 67.3% (34.5% +22.5% + 10.3%) and 63.8% (32.6% + 21.5% + 9.7%) of the time felt they had no mutual understanding in the events of a change in treatment plan and no discussion mechanism to maintain patient safety at least sometimes respectively. On the other hand 28.7% (7.9% + 20.8%) of the participants always or usually did feel they had the same understanding on patient’s care and 28.4% (6.9% + 21.5%) take in to account each other’s schedule when making plans to treat a patient together. In addition, around quarter, 25.6% (10.1% + 15.5%, and around one third, 32% (8.8% + 23.2%), of them always or usually felt they could openly exchange information or opinion about matters related to work and did help each other. Whereas, 29% (22.1% + 6.9%) did not listen to each other and 41.4% (27.5% + 13.9%) did not feel they received correct information or advice (Table 3).

**Table 3:** Frequency of perceived openness and sharing of information items during nurse-physician communication among nurses and physicians working in public Hospitals of Jimma Zone, Southwest Ethiopia, 2014 (n = 466).

Openness & sharing of information subscale item score(α = 0.91)	Always		Usually		Sometimes		Rarely		Never	
	No	%	No	%	No	%	No	%	No	%
In the event of a change in treatment plan, the nurse and the physicians have a mutual understanding	46	9.9	106	22.7	161	34.5	105	22.5	48	10.3
The nurse and physicians discuss mechanism to maintain patient safety	58	12.4	111	23.8	152	32.6	100	21.5	45	9.7
The nurse & the physicians have the same understanding on patient's care	37	7.9	97	20.8	148	31.8	107	23.0	77	16.5
The nurse & the physicians take into account each other's schedule when making plans to treat a patient together	32	6.9	100	21.5	150	32.2	113	24.2	71	15.2
The nurse & the physicians can openly exchange information or opinion about matters related to work	47	10.1	72	15.5	160	34.3	115	24.7	72	15.5
The nurse and the physicians show concern for each other when they are very tired	74	15.9	145	31.1	151	32.4	69	14.8	27	5.8
The nurse and the physicians help each other	41	8.8	108	23.2	172	36.9	104	22.3	41	8.8
Physicians and nurse listen to each other	50	10.7	116	24.9	165	35.4	103	22.1	32	6.9
Receiving correct information or advice	30	6.4	82	17.6	161	34.5	128	27.5	65	13.9

#### Perceived level of nurse-physician communication in patient care

The factor analysis of perceived level of nurse-physician communication measured by the two communication sub-scales showed the total variance explained 45.77% for perceived professional respect and satisfaction and 9.32% for perceived openness and sharing of patient information during nurse-physician communication (Table 4).

**Table 4:** Eigen values and the percentage of variance associated with each two components of communication sub-scales among nurses and physicians working in public hospitals of Jimma zone, 2014 (n = 466).

Components name	Eigen Values	Percentage of explained variance	Accumulated percentage of explained variance
Perceived professional respect and satisfaction	8.7	45.77	45.77
Perceived openness and sharing of patient information	1.77	9.32	55.09

Extraction Method: Principal Component Analysis

**Fig 2:**
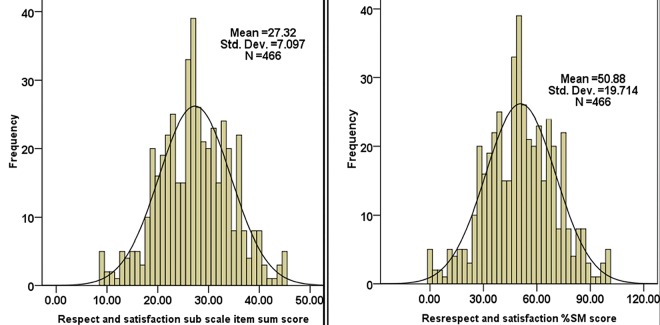
This is Perceived professional respect and satisfaction mean and maximum scale percentage mean scores in patient care among nurses and physicians working in public Hospitals of Jimma Zone, Southwest Ethiopia, 2014 (n = 466). * % SM score is the Standardized score as the percentage of possible maximum scale score and it lies between 0 and 100.

**Fig 3:**
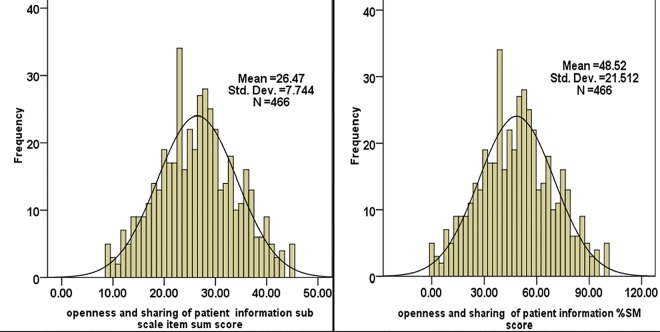
This is Perceived openness and sharing of information mean and maximum scale percentage mean scores in patient care among nurses and physicians working in public Hospitals of Jimma Zone, Southwest Ethiopia, 2014 (n = 466). * (%SM) is the Standardized score as the percentage of possible maximum scale score, and it lies between 0 and 100.

As shown in figure the perceived professional respect and satisfaction during nurse-physician communication had mean and maximum scale percentage mean score of 27.32 ± 7.1(Fig 2 left) and 50.88 ± 19.7% (Fig 2 right) respectively. The result on the perceived openness and sharing of information during nurse-physician communication showed mean and maximum scale percentages mean score of 26.47 ± 7.74 (Fig 3 left) and 48.52 ± 21.51% (Fig 3 right) respectively.

### Results of independent sample t-tests

Mean scores were compared between professional category, hospital category and sex using independent sample t-test in relation to the two communication sub scales. In the perceived respect and satisfaction on communication scale, the physicians’ mean of 28.8 (SD = 6.9) was significantly higher than the mean of nurses’ (mean = 26.6, SD = 7.1) at t = -3.3 and p<0.001. Similarly mean of nurses and physicians working in district/non-teaching hospitals (29.23 ± 5.79) was significantly higher than those working in teaching hospital (27.05 ± 7.23) with a p-value of 0.03. But there were no significant mean difference seen in sex (Table 5). Nurses and physicians who were working in district hospitals had more perceived openness and sharing of patient information (mean = 30.09±6.96) than those working in referral hospital (mean = 25.96±7.72) at t = 3.7and P = 0.002. Females had higher perceived openness and sharing of patient information (mean = 27.4±7.16) than male (mean = 25.93±8.02) at t = -2.04, p = 0.048) (Table 6).

**Table 5:** Independent sample t-test showing perceived nurse-physician communication as measured by respect and satisfaction in patient care among nurses and physicians working in public Hospitals of Jimma zone, 2014(n = 466).

Variables category		Perceived Respect and Satisfaction					
		N	Mean ± SD	T-tests	P-value	95% CI mean Difference	
						Lower	Upper
Profession category	Nurse	313	26.6 ± 7.1	-3.3	**0.001**	-3.62	-.89
	Physician	153	28.8 ± 6.9				
Hospital category	District/none teaching	57	29.23 ± 5.79	2.18	**0.03**	.217	4.14
	Referral/teaching hospital	409	27.05 ± 7.23				
Sex	Male	295	27.42 ± 7.30	0.43	0.68	-1.06	1.63
	Female	171	27.13 ± 6.74				

**Table 6:** Independent sample t-test showing perceived nurse-physician communication as measured by openness and sharing of information among nurses and physicians working in public Hospitals of Jimma zone, 2014 (n = 466).

Variable category		Perceived openness and sharing of information						
		N	Mean	Std. Dev.	T-test	P-valve	95% CI mean Difference	
							Lower	Upper
Profession category	Nurse	313	26.25 ± 7.95		-0.86	.39	-2.16	0.84
	Physician	153	26.9 ± 7.3					
Hospital category	District/non-teaching	57	30.09 ± 6.96		3.07	**0.002**	2.01	6.25
	Referral/teaching hospital	409	25.96 ± 7.72					
Sex	Male	295	25.93 ± 8.02		-2.04	**0.048**	-2.89	-0.06
	Female	171	27.4 ± 7.16					

### Results of ANOVA analysis

The ANOVA result showed that there was no mean difference seen among work experience groups in both perceived nurse-physician communication sub-scales. In the educational qualification category specialists and staff general practitioner physicians mean score of 30.43 (SD = 6.7) was significantly higher than the mean score for diploma holder nurses (mean = 26.05, SD = 6.86) at p<0.001 regarding professional respect and satisfaction with nurse-physician communication in patient care. But in the openness and sharing of patient information there was no significant difference among study participants’ educational qualification (Table 7).

**Table 7:** Multiple comparison ANOVA table of educational qualifications and perceived nurse-physician communication as measured by two communication scale among nurses and physicians working in public Hospitals of Jimma zone, 2014.

Communication Sub -Scales	Educational qualification category	N	Mean	Std. Dev.	F-statics	P -value	95% CI for Mean	
							Lower Bound	Upper Bound
Perceived professional respect and satisfaction on communication	Diploma nurse (**reference**)	196	**26.05**	6.86	6.35	**.000**	25.08	27.01
	BSc nurse	117	27.46	7.35			26.11	28.81
	Specialist and General Practitioner(staff)physician	61	**30.43**	6.65			28.72	32.13
	Resident physician (student)	92	27.77	6.94			26.34	29.21
	Total	466	27.32	7.1			26.67	27.96
Perceived openness and sharing of patient information on communication	Diploma nurse(**reference)**	196	26.24	7.66	1.76	0.15	25.16	27.32
	BSc nurse	117	26.27	8.47			24.71	27.82
	Specialist and General Practitioner(staff)physician	61	28.54	7.67			26.58	30.51
	Resident physician (student)	92	25.83	6.85			24.41	27.25
	Total	466	26.47	7.74			25.7607	27.1706

### Predictors of perceived nurse—physician communication in patient care

In the nurse-physician communication factor description, the top six factors described strongly agreed by nurses and physicians were absence of forum regarding nurse-physician communication (33.5%), frequent supply shortage in units (31.8%), malfunctioning of equipment in units (29.2%), lack of shared vision between nurses and physicians in hospitals (24.2%), lack of role and responsibility differentiation of nurses and physicians in hospitals (24%) and unfavorable attitude toward other profession (22.1%).

From perceived nurse-physician communication factors generated in the factor analysis the first component which was organizational factors explained 38.52% of the total variability and 58.1% was explained by the three components (Table 8). The mean (21.9 ± 5.4) and %SM score (66.26%) of organizational related factors were higher than personal behavior related individual factor and work attitude related individual factors (Table 9).

**Table 8:** Eigen values and the percentage of variance associated in the three nurse-physician communication factors among nurses and physicians working in public hospitals of Jimma zone, 2014 (n = 466).

Components named	Eigen values	Percentage of explained variance	Accumulated percentage of explained variance
Organizational factors	6.16	38.52	38.52
Work attitude individual factors	2.00	12.53	51.05
Personal behavior individual factors	1.13	7.05	58.10

Extraction method: principal component analysis.

**Table 9:** Mean and %SM scores for factors related to perceived level of nurse-physician communication in patient care among nurses and physicians working in public hospitals of Jimma Zone, Southwest Ethiopia, 2014 (n = 466).

Predictor sub scales or latent scales	Raw mean & %SM score	Nurse & physician
Organizational related factors	Mean score ± SD	21.9 ± 5.4
	%SM*	66.26
Work attitude related individual factors	Mean score ± SD	18.08 ± 5.59
	%SM*	50.34
Personal behavior related individual factors	Mean score ± SD	13.25 ± 3.64
	%SM*	57.82

*(%SM) is the Standardized score as the percentage of possible maximum scale score, and it lies between 0 and 100, SD = standard deviation.

### Results of linear regression analysis

Regression model was built in both bivariable and multiple variable linear regressions in order to find the significant predictors for the two nurse-physician communication subscales.

#### Predictor of perceived respect and satisfaction during nurse-physician communication in the bi-variable and multiple linear regression analysis

Thirteen predictors were entered independently to see their independent effect on respect and satisfaction. Variables with p-valve ≤ 0.25 in bi-variable analysis were entered in the final model (Table 10). Of these variables in the final model using the entered method age, current salary and organizational factors were significant predictors of perceived respect and satisfaction with nurse physician communication and explains 8.1% of the variability in the dependent variable. Current salary of participants has a positive effect on perceived respect and satisfaction with nurse-physician communication in patient care, whereas age of participants and organizational factors have a negative effect (Table 11).

**Table 10:** Bivariate linear regression predicting perceived respect and satisfaction during nurse-physician communication in patient care among nurses and physicians working in public Hospitals of Jimma zone, 2014(n = 466).

Predictor variables		Unstd. Coeff.		Std. Coeff.	p-value	95% CI for B	
		β	Std. Error	β		Lower Bound	Upper Bound
Hospital category	Referral	-0.16	0.14	-0.051	0.27	-0.44	0.12
	District	0.157	0.141	0.051	0.268	-0.12	0.44
Profession category	Physician	0.37	0.097	0.174	<0.001**	0.18	0.56
	Nurse	-0.371	0.097	-0.174	<0.001**	-0.562	-0.18
Sex category	Female	-0.16	0.096	-0.075	0.11*	-0.34	0.03
	Male	0.155	0.096	0.075	0.108*	-0.03	0.34
Age in years		-0.01	0.007	-0.056	0.23*	-0.02	0.01
Marital status category	Single	0.078	0.09	.039	0.404	-.105	.261
	Ever married	-0.08	0.09	-0.04	0.40	-0.26	0.11
Length of service in years		-0.01	0.01	-0.07	0.12*	-0.03	0.003
Educational qualification	Diploma nurse	-0.37	0.092	-0.18	<0.001**	-0.55	-0.191
	BSc nurse	0.05	0.11	0.02	0.650	-.162	0.26
	Specialist and staff GP	0.5	0.14	0.17	<0.001**	.232	0.77
	Resident	0.16	0.12	0.06	0.18*	-0.07	0.39
Working unit category	Outpatient						
	Inpatient	-0.13	0.1	-0.06	0.18*	-0.33	0.06
Position category	Without responsibility	-0.06	0.15	-0.02	0.68	-0.36	0.24
	With responsibility	0.06	1.15	0.02	0.68	-0.24	0.36
Current salary		0.001	.00	0.17	<0.001**	0.00	**<0.001**
Work attitude factor		-0.02	0.05	-0.02	0.74	-0.11	0.08
Personal behavior factor		-0.05	0.05	-0.05	0.29	-0.14	0.04
Organizational factor		-.005	0.05	-0.005	0.047**	-0.1	0.09

*: Candidate for multivariable model

**: Significant association in bivariable linear regression

**Table 11:** Multiple variables linear regression predicting perceived respect and satisfaction with nurse-physician communication in patient care among nurses and physicians working in public Hospitals of Jimma zone, 2014(n = 466).

Predictor variables		Unstd. Coeff.		Std. Coeff.	T	p-value	95% CI for B	
		β	Std. Error	β			Lower Bound	Upper Bound
	(Constant)	0.3	0.2		1.50	0.134	-0.09	0.69
	Age in year	-0.02	0.01	-0.16	-3.2	0.001**	-0.04	-0.01
	Current salary	0.01	0.00	0.24	4.76	0.00**	0.002	0.03
	Organizational factors	-0.09	0.04	-0.11	-2.34	0.020**	-0.17	-0.02

Adjusted R^2^ = 0.081, Maximum VIF = 1.41, Minimum VIF = 1.04

**:significant for multivariable linear regression

#### Predictor of openness and sharing of patient information during nurse-physician communication in the bi-variable and multiple linear regression analysis

Regarding respect and satisfaction thirteen variables were entered independently to see their independent effect on openness and sharing of patient information. Then all variables in bivariate analysis with p< = 0.25 (Table 12) were entered to the final model through entered methods. Sex was positively associated with perceived openness and sharing of patient information during nurse physician communication, whereas hospital category, work attitude individual factor and organizational factors were negatively associated. These predictors explained 10.4% variability of openness and sharing of patient information during nurse-physician communication.

**Table 12:** Bivariate linear regression predicting perceived openness and sharing of patient information during nurse-physician communication in patient care among nurses and physicians working in public Hospitals of Jimma zone, 2014(n = 466).

Predictor variables		Unstd. Coeff.		Std. Coeff	P	95% CI for B	
		β	Std. Error	β		Lower Bound	Upper Bound
Hospital category	Referral	-0.51	0.14	-0.17	0000**	-0.78	-0.23
	District	0.51	0.14	0.17	0.00**	0.23	0.78
Profession category	Physician	-0.08	0.1	-0.04	0.45	-0.27	0.12
	Nurse	0.08	0.1	0.04	0.45	-0.12	0.27
Sex category	Female	0.29	0.1	0.14	.003**	0.1	0.47
	Male	-0.29	.095	-0.14	.003**	-0.47	-0.1
Age in years		0.001	0.01	0.01	0.84	-0.01	0.02
Marital status category	Single	-0.09	.093	-0.05	0.32	-.028	0.09
	Ever married	0.09	0.09	.046	0.32	-0.09	0.28
Length of service in years		0.004	0.01	0.023	0.61	-0.01	0.02
Educational qualification	Diploma nurse	0.12	0.09	0.057	0.22*	-0.07	0.30
	Bsc nurse	-0.062	0.107	-0.027	0.56	-0.27	0.15
	Specialist and staff GP	0.11	0.14	0.04	0.44	-0.16	0.38
	Resident	-0.18	0.12	-0.07	0.12*	-0.41	0.05
working unit category	Outpatient	0.07	0.1	0.03	0.5	-0.13	0.26
	Inpatient	-0.07	0.1	-.03	0.5	-0.26	.013
Position category	Without responsibility	-0.07	0.15	-0.02	0.63	-0.37	0.23
	With responsibility	0.07	0.15	0.02	0.63	-0.23	0.37
Current salary		-0.0014	0.00	-0.03	0.59	0.00	0.00
Work attitude factors		-0.023	.008	-0.13	.006**	-0.04	-0.01
Personal behavior factors		-0.022	.046	-.022	.633	-0.11	0.07
Organizational factors		-0.021	0.01	-0.11	0.017**	-0.037	-0.004

**: significant association in Bivariable linear regression

*: Candidate for multivariable linear regression

Nurses and physicians who were working in referral hospital had 0.44 decrease in their perceived openness and sharing of patient information during nurse-physician communication than those working in district level hospitals (p = 0.002). Being female had 0.23 increase in perceived openness and sharing patient information during nurse-physician communication in patient care than male (p = 0.017). For a unit increase in perceived work attitude individual factors score, the perceived openness and sharing of patient information decreased by an average of 0.08 (p = 0.037). Unit increase in perceived organizational factor score decreased openness and sharing of patient information during nurse-physician communication by 0.1, at p = 0.025 (Table 13).

**Table 13:** Multiple variables linear regression predicting perceived openness and sharing of patient information during nurse-physician communication in patient care among nurses and physicians working in public Hospitals of Jimma zone, 2014(n = 466).

Predictor variables		Unstd. Coeff.		Std. Coeff.	T	p-valve	95% CI for B	
		β	Std. Error	β			Lower Bound	Upper Bound
	(Constant)	0.30	0.14		2.18	0.03	0.03	0.57
	Hospital category(referral)	-0.44	0.14	-0.14	-3.15	0.002**	-0.71	-0.17
	Sex category(Female)	0.23	0.1	0.11	2.4	0.017**	.04	0.42
	Work attitude individual factors factor	-0.08	0.05	-0.08	-1.80	0.037**	-0.17	0.01
	Organizational factor	-0.10	0.05	-0.10	-2.24	0.025**	-0.19	-0.01

Adjusted R^2^ = 0.104, Maximum VIF = 1.039, Minimum VIF = 1.009

**: significant for multivariable linear regression, male and district hospitals were reference groups

## Discussion

In hospital setting, the common project that nurses and physicians share is serving patients. To achieve desired quality of patient outcome having the right nurse-physician communication is an important strategy and brings solutions for collaborative patient care by reducing major risk factors to patient safety such as lack of critical information, misinterpretation of information, medication errors and others [8, 23]. But the two professionals also have different perspectives on their interprofessional communication and factors affecting their communication.

In this study the result showed that nurses' and physicians' perceptions score of their interprofessional communication mean score were: 50.88% in perceived professional respect and satisfaction and 48.52% in perceived openness and sharing of patient information during nurse-physician communication in patient care. The scores indicate that nurse-physician interprofessional communication were closest to the standard mean (%SM = 50), which shows that perceived level of nurses and physicians communication has attention seeking gap in their communication level. Hence, the two scales represent the prioritized point of focus for nurse-physician communication intervention.

Although scores were closest to 50% in both communication sub-scales perceived communication level was less among nurses than physicians. This finding is consistent with previous studies done in Texas, VHA West Coast and West Indies which showed that physicians’ communication score was better than nurses’ [16, 20, 25, 33]. In contrast; nurse had higher mean score than physicians for professional respect in Egypt [27] and sharing of patient information in USA [26]. This discrepancy may be due to nurse’s better autonomy on their practice in Egypt and USA than this study area. If there is no professional respect and proper patient information sharing between nurses and physicians interprofessional communication, disregard between the professionals will occur and the health care team communication in turn will be affected which further affects the quality of care and patient outcomes.

In addition this study showed that higher mean scores were found in the perceived respect and satisfaction among participants with higher educational level. Specialists had higher mean score in the perceived respect and satisfaction score than the others. The finding is supported by higher education levels associated with greater perceptions of communication done in Midwestern hospitals of Korea [9]. This finding is likely explained by increasing role expectations as educational level increases as compared to others.

The result of our study revealed that mean score of perceived professional respect and satisfaction (p = 0.03) and mean score of perceived openness and sharing of patient information (p = 0.002) among nurses and physicians working in referral hospital were less than those who were working at district level hospitals. These differences could be district hospitals may have less patient flow than referral; however the referral hospital serves for clients who are coming from different health facilities by referral system which may add the burden to those nurses and physicians who are working in it and could affect openness and information sharing which might requires time to share among nurses and physicians. Moreover, in JUTH since there were large number of resident physicians, medical interns and nurse students and there was no well-defined communication channels which may affect the level of communication between nurses and physicians.

Our study identified a significantly higher perceived nurse-physician communication in openness and sharing of patient information dimension by female than male during nurse-physician communication (*p =* 0.017). This finding is supported by a study done in Iran where the mean score of openness was higher among female nurses [28].

In the current study increasing age had negative relation with respect and satisfaction among the study participants (p = 0.001). This finding was consistent with a study done in Japan among doctors where older age doctors had negative perception of interprofessional collaboration which could affect their communication [7], but this finding is not consistent with the study done in Iran which showed no difference in perception of nurse-physician communication among different age groups [28]. This difference might be due to the fact that most of the participants of the current study were young.

In this study participants with higher monthly salary had a significant higher perceived respect and satisfaction during nurse-physician communication (p<0.001). This could indicate that relatively better payment might facilitate good nurse-physician communication.

Almost one third of the study participants strongly agreed that absence of forum regarding nurse- physician communication in their institutions was one of the major factors contributing to nurse-physician miscommunication in patient care. This shows the need for interprofessional forum in hospitals that can improve outcomes of patients, nurse-physician interprofessional relationships and collaboration. Without interprofessional forum, health professionals tend to carry on working without realizing the advantages of interprofessional collaboration [7].

Moreover, in nurse-physician communication organizational factors were the first rated factors (66.26%) than personal behavior related individual factors and work attitude individual factors. All the three factors were scored above 50% which showed that these factors affect the perceived nurse-physician communication in patient care. Previous studies conducted in Belgium, Japan, Connecticut and South Nigeria however, identified factors such as poor interpersonal communication skills, roles misunderstanding, poor work attitude to the other profession, personal behavior and gender were identified as potential barriers to effective nurse-physician communication [7, 11, 13, 34].

Our study also identified that organizational factors affect both perceived respect and satisfaction (p = 0.02) and perceived openness and sharing of patient information (p = 0.025) during nurse- physician communication while perceived work attitude individual factors affect perceived openness and sharing of patient information (p = 0.37). The finding is supported by study done in USA which suggests that individual work attitude and organizational factors influence the character of a communication [35].

The usefulness of dealing issues regarding nurse-physician communication and influencing factors found to help enhance nurse-physician communication in the studied hospitals.

### Limitation of the study

The findings in this report were subjected to respondents’ discussion with their colleagues to answer the question that might result in social desirability bias. In addition, since most physicians in the teaching hospital were resident students who came from different part of the country for education this might under estimate the result. However, efforts were made trying in pretesting questionnaires and involving both nurse and physician as study participants.

### Implications for practice

As shown above and mentioned by different literatures understanding level and factors of perceived nurse-physician communication are important to fill gap and strengthen effective nurse-physician communication. The result of current study reflects the usefulness of promoting nurse-physician communication and factors to improve nurse-physician communication in the studied hospitals. As nurse-physician communication become effective we can gain good quality patient care and better patient outcome by preventing miscommunication mishaps in the hospital.

## Conclusions and Recommendations

### Conclusions

As this study assessed the perceived nurse-physician communication and associated factors, it is an essential element for collegiality and development of both professions. Ineffective communication between nurses and physicians resulted in delaying care, extending the length of a patient's stay in the hospital, and causing patient injury and death. Generally, the following points were concluded from the current study:

- The study populations in Jimma zone public hospitals were younger work force with a mean age of 28.95±6.82years.
- The overall perceived level of nurse-physician communication was almost 50% for both perceived professional respect and satisfaction and perceived openness and sharing of patient information on nurse-physician communication which can be the prioritized point of focus for nurse-physician communication intervention.
- Around 1/3^rd^ of the nurses and physicians were angry and frustrated after nurse-physician interaction and around half of them did receive correct information sometimes, rarely or never regarding patient care.
- Though significant proportion of nurses and physicians did show concern for each other, around 1/3^rd^ of them did not have mutual understanding in patient treatment plan and each other’s’ schedule.
- Generally the communication level of nurses was lower than the physicians’ score.
- Mean score of perceived respect and satisfaction and mean score of perceived openness and sharing of information were higher among nurses and physicians working in district/non-teaching hospitals.
- Age, current monthly salary and organizational factors were the potential predictors for perceived respect and satisfaction while sex, working hospital category, work attitude individual factors and organizational factors were predictors of perceived openness and sharing of patient information in nurse-physician communication during patient care.
- Salary has a positive effect on perceived respect and satisfaction whereas age and organizational factors have a negative effect.
- This finding showed nurse-physician communication needs attention for providing a mutually planned and understood patient care.

### Recommendations

The study showed that nurses and physicians need to assess their current state of nurse–physician communication in their institution. Finally, based on study findings the following points were suggested for respective groups.

#### Recommendations for nurses and physicians

As part of professional obligation and for better outcome of patients, nurses and physician should discuss about their communication level while giving care to the patient, communicate openly, in mutual professional respect, and share patient’s information. Moreover, these professional need play a vital role in creating smooth and a well-defined communication channel.

#### Recommendations for hospital management bodies

The Hospital management should have usual nurse-physician staff meetings regarding nurse-physician communication in patient care, support nurses and physicians to communicate openly and frankly, discuss on the impact of nurse-physician communication in patient care and health care quality given in their institution and need to make sure that equipment are well functioned in the units. Forum for nurses and physicians need to be scheduled on a certain defined time interval. In addition, in collaboration with the government health offices at different levels the hospitals need to device strategies used to facilitate nurse-physician communication.

#### Recommendations for nursing and medical school’s curriculum

Support the schools of nursing and medical schools to develop curricula regarding nurse-physician communication skills, better to organize nursing and medical student’s team which controls the flow of communication between them.

#### Recommendations for further researchers

Study the impact of nurse-physician communication on staff, patient, organizational, and financial outcomes and hospital patient care quality.

## Supporting Information

S1 File Nurse-physician communication SPSS data set. (SAV)
